# Branching patterns of olivocerebellar axons in relation to the compartmental organization of the cerebellum

**DOI:** 10.3389/fncir.2013.00003

**Published:** 2013-02-04

**Authors:** Hirofumi Fujita, Izumi Sugihara

**Affiliations:** ^1^Department of Systems Neurophysiology, Tokyo Medical and Dental University Graduate SchoolTokyo, Japan; ^2^Systems Neurobiology Laboratories, The Salk Institute for Biological StudiesLa Jolla, CA, USA

**Keywords:** climbing fibers, collaterals, aldolase C, zebrin, lobules, compartments, somatotopical representation

## Abstract

A single olivocerebellar (OC) axon gives rise to about seven branches that terminate as climbing fibers (CFs). Branching patterns of an OC axon, which are classified into local, transverse, and longitudinal types, are highly organized, in relation to the longitudinal molecular (aldolase C or zebrin II) compartmentalization and the transverse lobulation of the cerebellum. Local branching is involved in forming a narrow band-shaped functional subarea within a molecular compartment. On the other hand, transverse and longitudinal branchings appear to be involved in linking mediolaterally separated molecular compartments and rostrocaudally separated lobular areas, respectively. Longitudinal branching occurs frequently between equivalent molecular compartments of specific combinations of lobules. These combinations include lobule V-simple lobule and crus II-paramedian lobule in the pars intermedia and hemisphere, and lobules I–V and lobule VIII in the vermis. The longitudinal branching pattern not only fits with mirror-imaged somatosensory double representation of the body in the pars intermedia, but it also suggests a general rostrocaudal link exists for the whole cerebellum across the putative rostrocaudal boundary in lobule VIc-crus I. Molecular compartments of the cerebellar cortex originate from the Purkinje cell (PC) clusters that appear in the late embryonic stage, when the immature OC projection is formed. Some clusters split rostrocaudally across crus I during the development of cortical compartments, which would result in longitudinal branching of OC projection across crus I. Supposing that the branching pattern of OC axons represents an essential organization of the cerebellum, longitudinal branching suggests a functional and developmental links between the rostral and caudal cerebellum across lobule VIc-crus I throughout the cerebellar cortex.

## Introduction

The cerebellar cortex is subdivided two ways: transversely by its lobular folding and longitudinally by PC subset compartments that are defined by the expression patterns of certain molecules, such as aldolase C or zebrin II (Brochu et al., 1990; Ahn et al., 1994). While the lobular and compartmental organizations are complex, a concrete functional localization is founded on these organizations in the cerebellar cortex (Nieuwenhuys et al., 2008).

Involvement of a cerebellar subarea in specific functions is thought to be largely dependent on regional differences in afferent and efferent connectivity. Climbing fiber (CF) afferents, which originate exclusively from the inferior olive (IO) as the OC projection, are particularly well-organized in a topographic sense. CFs that arise from a specific IO subdivision form synapses onto PCs in a particular aldolase C compartment that are about 0.1–0.5 mm in mediolateral width but elongated in the longitudinal direction (Voogd et al., 2003; Sugihara and Shinoda, 2004). In turn, PCs located within each compartment provide a highly convergent projection to specific regions of the cerebellar and vestibular nuclei (Voogd and Bigaré, 1980; Buisseret-Delmas and Angaut, 1993; Apps and Garwicz, 2005; Sugihara et al., 2009).

While the PC projection from the cortex is highly convergent, afferent projections to the cortex, CFs as well as mossy fibers (MFs), are characterized by their divergent branching patterns (Sugihara et al., 1999, 2001, 2009; Wu et al., 1999). Since CFs and MFs are the two main afferents of the cerebellar cortex, the branching patterns of CF and MF axons (in other words, the positional relationships between CF and MF terminals that originate from a single axon) are supposed to be tightly involved in the functional organization of the cerebellum. Since a CF is supposed to significantly affect the activity of an individual PC by its one-to-one innervation, topographic organization of the olivocerebellar (OC) projection, including the branching pattern of individual axons, is particularly critical in determining the functional organization of the cerebellum.

The branching patterns of cerebellar afferent and efferent axons have been analyzed clearly by reconstructing single axons as well as by other methods, which we review in this article, mainly focusing on OC axons.

## Branching of individual OC axons

The fact that the number of IO neurons is smaller than that of PCs (1:7 in the rat, Schild, 1970) implies a branching of OC axons, since a CF, terminal portion of the OC axon, generally projects to a PC in one-to-one relationship (Ramón y Cajal, 1911). Local branching of an OC axon in the cerebellar cortex has been shown in Golgi staining preparation in monkey (Fox et al., 1969). Local branching of an OC axon has also been demonstrated by electrophysiological recording of the “climbing fiber reflex” between lobule III and V in the cat (Faber and Murphy, 1969). Later, single axonal reconstruction has shown that an OC axon gives rise to several types of branches in the rat (Sugihara et al., 1999). One OC axon gives rise to about seven “thick” branches that terminate as CFs, and about nine “thin” collaterals that terminate mainly in the granular layer and the deep cerebellar nucleus (DCN) in the rat (Van der Want et al., 1989). Granular layer collaterals terminate in a similar cortical area where some thick branches terminate in the molecular layer as CFs. Nuclear collaterals terminate in a topographically related small area in the cerebellar nuclei (reviewed in Sugihara, 2011). We will focus on “thick” branches that terminate as CFs in the rest of this article.

## Classification of spatial pattern of branching: local, transverse, and longitudinal branching

### Local branching

**Figure 1:**
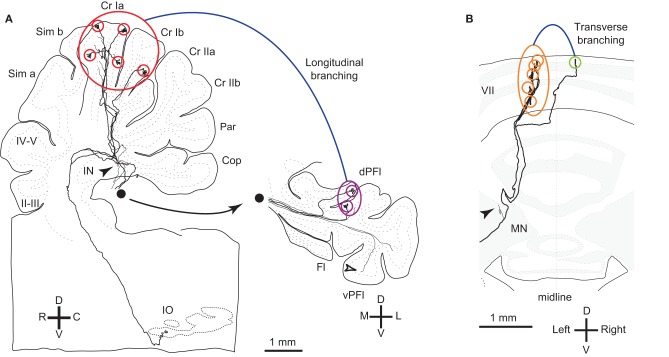
**Reconstructed OC axons showing different types of branching. (A)** Reconstructed rat OC axon that terminated in crus I and paraflocculus with five (red circles) and two (purple circles) CFs, respectively, indicating longitudinal and local branching. This axon also had thin collaterals terminating in the granular layer (open arrowheads) and cerebellar nuclei (filled arrowheads). This axon was reconstructed in our previous study (Sugihara et al., 1999). Filled circle shows continuation of axons from the white matter of the paraflocculus. **(B)** Reconstructed rat OC axon that terminated mainly in the left lobule VII (orange circles) but also had a transcommissural transverse branch (yellow-green circle) (same axon as shown in Figure 4B of Sugihara et al., 1999). Abbreviations in this and subsequent figures, I–X, lobules I–X; a-d, sublobules a-d; AIN, anterior interposed nucleus; C, caudal; CF, climbing fiber; Cop, copula pyramidis; Cr I, crus I of ansiform lobule; Cr II, crus II of ansiform lobule; D, dorsal; das, dorsal acoustic stria; DCN, deep cerebellar nuclei; dPFl, dorsal paraflocculus; Fl, flocculus; fp, floccular peduncle; IN, interposed nucleus; IO, inferior olive; L, lateral; LN, lateral cerebellar nucleus; M, medial; MN, medial cerebellar nucleus; p, sublobule p; Par, paramedian lobule; PC, Purkinje cell; pf, primary fissure; PIN, posterior interposed nucleus; R, rostral; Sim, simple lobule; V, v−, ventral; vPFl, ventral paraflocculus.

**Figure 2:**
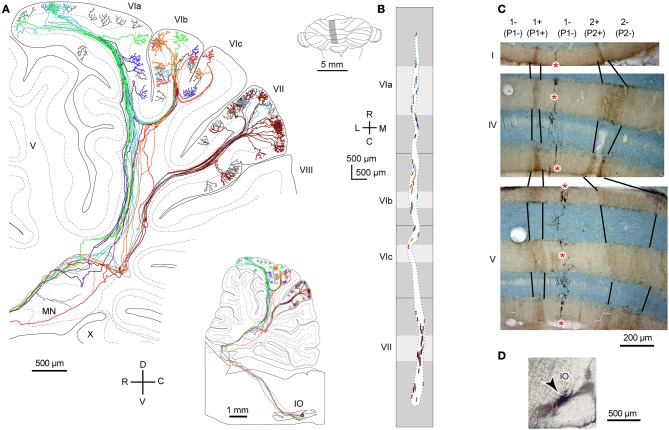
**Local branching terminating in a narrow longitudinal band-shaped area. (A)** Six axons that were labeled with a small injection of biotinylated dextran amine to the IO were reconstructed. The inset shows the entire trajectory of these axons. **(B)** Distribution of labeled CFs of axons in **(A)** (six colors) and other axons that were not reconstructed (gray) were plotted on the unfolded cerebellar cortex. Light and dark gray areas indicate apical and fissural parts of the lobule. **(C)** Double labeling of CFs (in black, asterisks) and aldolase C (brown) in cerebellar sections. CFs labeled by a localized injection of biotinylated dextran amine to the IO were distributed in a narrow band-shaped area that occupied a portion of a single aldolase C compartment. **(D)** Injection site of biotinylated dextran amine in the rat IO. **(A)** and **(B)** are cited from Sugihara et al. (2001). **(C)** and **(D)** are based on the data in Sugihara et al. (2004). 1+, 1−, 2+, names of aldolase C compartments according to the nomenclature of Sugihara and Shinoda (2004), which are equivalent to “P1+,” “P1−,” and “P2+” as often used in ligature (Brochu et al., 1990; Apps and Hawkes, 2009).

We classify branching patterns of OC axons into local, transverse, and longitudinal types in this article (Figure 1). Multiple CFs that originate from an OC axon terminate in a single lobule or neighboring lobules. These CFs are often distributed in a narrow longitudinal band-shaped area (0.1–0.3 mm wide in rats). We designate this type of branching as local branching. CF termination of local branching of OC axons that originate from a small subarea in the IO share the same narrow longitudinal band-shaped area (Figures 2A and B). This narrow band-shaped area usually occupies a small sub-compartment within a single longitudinal compartment defined by the aldolase C expression pattern (Figures 2C and D; refer to (Voogd et al., 2003; Sugihara and Shinoda, 2004; Sugihara, 2011) for aldolase C compartments). Twenty-two out of thirty-four reconstructed OC axons had only local branching (Sugihara et al., 2001).

### Transverse branching

**Figure 3:**
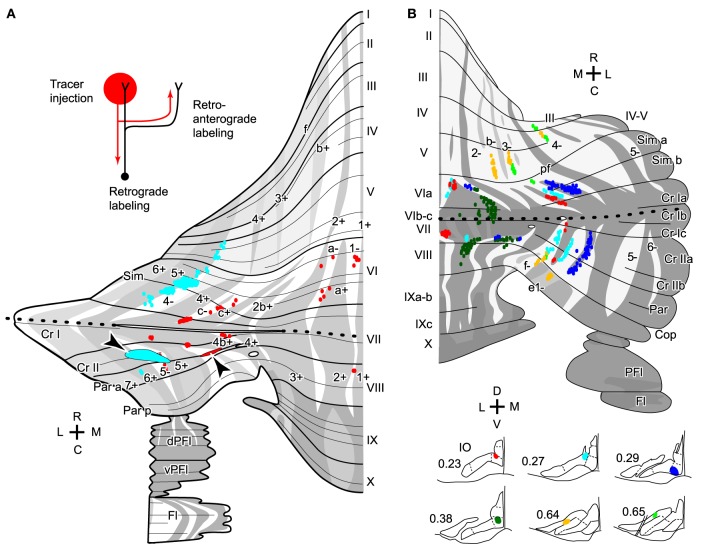
**Transverse branching of olivocerebellar axons. (A)** Mapping of CFs that were labeled retro-anterogradely by a biotinylated dextran amine injection in the cerebellar cortex in marmosets (arrowheads). While an injection in crus II in pars intermedia (red) labeled CFs in transversely separate compartments, indicating transverse branching, an injection in crus II in the hemisphere (cyan) labeled CFs only in the longitudinally separate areas, indicating presence of longitudinal branching but absence of transverse branching. Inset in the top left-hand corner illustrate retro-anterograde labeling. **(B)** Mapping of labeled CFs labeled by a small injection of biotinylated dextran amine into the mouse IO, mapped in the scheme of aldolase C compartments in the cerebellar cortex. Cases in which CFs were labeled in multiple mediolaterally separate compartments are summarized here, suggesting transverse branching. CFs are also distributed in longitudinally separate areas, suggesting longitudinal branching. Single axons were not reconstructed. **(A)** and **(B)** are based on the data in Fujita et al. (2010) and Sugihara and Quy (2007), respectively. Dotted lines indicate the rostrocaudal boundary.

Transverse branching of an OC axon was first noticed by electrophysiological recording of somatosensory responses between C1 and C3 zones, which correspond to aldolase C-negative (including lightly-positive) compartments in the medial and lateral pars intermedia, respectively, in lobule V in cats (Ekerot and Larson, 1982). However, CF labeling by tracer uptake from CF terminals of the same axon (designated retro-anterograde labeling, Figure 3A inset) has also shown transverse branching of OC axons between C1 and C3 zones in lobules III–V in rats (Voogd et al., 2003). We observed transverse branching of OC axons in multiple aldolase C-negative stripes in the vermis and pars intermedia in retro-anterograde OC axon labeling in the marmoset cerebellum (Figure 3A, orange). We define typical transverse branching as branching between unilateral and mediolaterally distinct compartments in the same or adjacent lobules. Although we did not see typical transverse branching in our samples of reconstructed single axons in rats (*n* = 34 axons, Sugihara et al., 2001), results from a prior study suggested that it, in fact, occurs (Sugihara and Shinoda, 2004). Following a small injection of biotinylated dextran amine in the IO labeled CFs often appeared in multiple mediolaterally separate compartments in the lateral vermis and pars intermedia (seen in roughly in half cases, Figures 5B,C, 6B, and 7A,B of Sugihara and Shinoda, 2004), suggesting transverse branching (Figure 3B).

OC axon branching between the nodulus and flocculus (Takeda and Maekawa, 1989; Sugihara et al., 2004) may also be considered an example of transverse branching. Transcommissural transverse branching, in which a branch terminates in the opposite side about the midline, has been observed in the vermis less frequently (Figure 1B; Sugihara et al., 1999).

### Longitudinal branching

**Figure 4:**
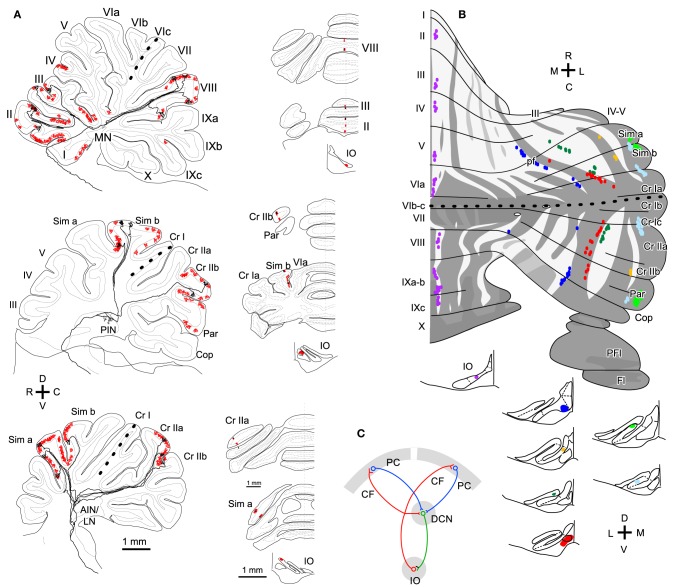
**Longitudinal branching of olivocerebellar axons. (A)** Reconstructed axon depicted with all other CFs labeled by a small injection of biotinylated dextran amine into the IO in three rats. Drawings of labeled CFs in coronal sections and injection sites in the IO were also shown (right-hand insets). Red color indicates labeled CFs that did not belong to the reconstructed axon. **(B)** Mapping of labeled CFs labeled by a small injection of biotinylated dextran amine into the mouse IO, mapped in the scheme of aldolase C compartments in the cerebellar cortex. Cases in which CFs were labeled only in longitudinally separate pair of equivalent compartments are summarized here, suggesting longitudinal branching. **(A)** and **(B)** are based on the data in experiments in Sugihara et al. (2001) and Sugihara and Quy (2007), respectively. **(C)** Schematic of the closed-loop-shaped modular organization between the IO, cerebellar cortex and DCN modified by the longitudinal branching of the OC axon.

Here we define longitudinal branching as branching to non-contiguous lobules in the cerebellar cortex. Longitudinal branching was first demonstrated with the CF reflex between lobule V and the paramedian lobule in the cat pars intermedia (Armstrong et al., 1973). A double retrograde labeling study has also shown longitudinal branching between lobule V and the paramedian lobule (Rosina and Provini, 1983). Branching is formed between C1 zones in lobule V and paramedian lobule, which are physiologically equivalent areas that both receive forelimb cutaneous inputs (Apps, 2000). Single axon reconstruction has shown that longitudinal branching occurs frequently (in 12 out of 34 axons) not only in the pars intermedia but also in the vermis and hemisphere (Figure 4A; Sugihara et al., 2001). Mediolateral positions of the termination areas of longitudinal branching are usually similar (Sugihara et al., 2001). Longitudinal branching is usually distinguished easily from local branching, which targets single or neighboring lobules, in terms of morphology of axonal trajectories (Figure 1A).

Molecular compartmentalization of the cerebellar cortex, which is visualized by immunostaining of aldolase C or some other molecules (Brochu et al., 1990), has an ultra-tight relationship with the longitudinal branching of OC axons (Voogd et al., 2003; Sugihara and Shinoda, 2004). The aldolase C expression pattern shows mediolateral merging and lateral shift in vermal lobule VIc and crus I, appearing to be a landmark for the center between the rostral and caudal cerebellum (designated as “rostrocaudal boundary,” Sugihara and Shinoda, 2004). Longitudinal branches often terminate in aldolase C compartments that have similar expression levels of aldolase C and are located at a similar mediolateral distance from the midline in the rostral and caudal cerebellum (Figure 4B; Sugihara and Shinoda, 2004; Sugihara and Quy, 2007). Even if these aldolase C compartments are not really continuous, targeting of longitudinal branches of an axon to these compartments indicates that they are paired or linked compartments (see Table 1 of Sugihara and Shinoda, 2004).

In regards to target lobules, the most typical pairs of lobules that receive longitudinal branches include (1) simple lobule and caudal lobule V vs. crus II and paramedian lobule in the pars intermedia and hemisphere, (2) lobules II–V vs. copula pyramidis in the pars intermedia, and (3) lobules II–V and lobule VIII in the vermis (Sugihara and Shinoda, 2004). These longitudinal branching patterns are consistent with the mirror-image organization about the “rostrocaudal boundary” positioned on crus I (Sugihara and Shinoda, 2004; Sugihara and Quy, 2007). Other longitudinal branching has been seen between lobule VIa-b and lobule IX in the vermis, between crus I and the paraflocculus in the hemisphere, and between the most lateral part of crus I and the flocculus (Sugihara et al., 2004). Thus, the longitudinal branching occurs not randomly but in specific pairs of lobules. This reflects the basic organization of the cerebellum.

## Projection patterns of PC axons

To relate branching patterns of single OC axons to cerebellar function requires looking at efferent PC projection patterns. A major question is whether PCs that receive branches of a single OC axon converge in their output projection. In relation to the local branching that terminates in a narrow band-shaped area, an electrophysiological study has shown that PCs located in such an area (one of the five subzones in “zone B” in the lateral vermis) generally project and converge to a distinct neuronal group in the lateral vestibular nucleus (Andersson and Oscarsson, 1978), which is equivalent to the DCN in receiving PC projections. An anatomical labeling study of multiple PCs indirectly supported the convergent PC projection from a band-shaped area (Sugihara et al., 2009). Furthermore, PCs that slightly separate in the mediolateral direction (but still in the same molecular compartment) terminate in slightly mediolaterally separate areas in the DCN. Therefore, the narrow longitudinal band-shaped area in the cerebellar cortex may represent the basic operational units of the cerebellum.

Concerning transverse branching of OC axons, Apps and Garwicz (2000) have shown that corticonuclear projections from C1 and C3 zones in lobule V, which receive forelimb cutaneous inputs, converge to the same area in the anterior interposed nucleus (AIN). Furthermore, depending on the receptive field (ulnar, radial, and ventral side of the forelimb), the projections from C1 and C3 zones converge on slightly different areas in the AIN. Retrograde PC labeling by localized injections of retrograde tracers in the AIN supports convergence of PC projections from C1 and C3 zones (Pijpers et al., 2005). In other areas, projections of PCs that receive transverse branches of OC axons have not been fully studied yet.

Concerning the longitudinal branching, PCs in separate lobules of the rostral and caudal cerebellum are simultaneously labeled by a localized injection of retrograde tracers in the DCN, indicating convergence of PC projections (Pijpers et al., 2005; Sugihara et al., 2009). Anterograde PC labeling has also shown that PCs located in the same (or paired) aldolase C compartment(s) in adjacent (crus IIa and IIb) or separate lobules (simple lobule and crus II) in the cerebellar cortex project to the same small area in the DCN (Sugihara et al., 2009).

In sum, experimental data generally support the idea that PCs that receive local, transverse, and longitudinal branches of a single OC axon make a convergent projection to the same small area in the DCN. It should be noted that the target area of nuclear collaterals of OC axons generally coincides with the target area of the PCs that are innervated by these OC axons (Sugihara, 2011). Thus, there is generally a closed-loop-shaped modular organization between small subareas within the IO, DCN, and cerebellar cortex connected by the topographic OC and corticonuclear projections (Pijpers et al., 2005; Sugihara, 2011), and by the nucleoolivary projection (Ruigrok and Voogd, 1990; Marshall and Lang, 2009). If branching divergence in the projections of OC axons and convergence in the corticonuclear PC projection are taken into account, the closed-loop-shaped modular organization needs to be modified; the loop is divided into multiple parallel loops in the cerebellar cortex (Figure 4C).

## Branching of mossy fiber axons

Although this article is focused on OC axons that terminate as CFs, we have also briefly looked at the MFs that constitute the other main afferent system in the cerebellar cortex. Axonal branching patterns of MF axons (Wu et al., 1999; Quy et al., 2011) are clearly different from that of OC axons or from PC axons. Basically, the stem MF axon runs transversely in the deep cerebellar white matter, giving rise to several branches at different mediolateral locations. While classifying branches into definite types can be difficult, many branches generally have secondary, tertiary, and further branches that can be of transverse, longitudinal, or local types (Quy et al., 2011) As a whole, rosette-shaped terminals of a MF axon (about 100 per axon, Wu et al., 1999; Quy et al., 2011) are distributed in many band-shaped or patch-shaped areas in multiple lobules. Although there is still some topographical pattern in each MF projection originating from a specific subarea of the precerebellar nuclei, it is not generally simple to relate branching patterns of MF axons to that of OC axons or from PC axons (Quy et al., 2011).

## Relationship to the functional organization of the cerebellum

### Local branching

OC axons originating from IO neurons located in close vicinity to each other project to the same or an overlapping narrow longitudinal band-shaped area (0.1–0.3 mm wide) with their local branches (Sugihara et al., 2004). We speculate that these neighboring IO neurons share input and thus have a similar responsiveness to stimuli and, consequently, PCs in a narrow longitudinal area have a similar responsiveness in their complex spike activity. Indeed, such band-shaped areas, also called “microzones,” have been shown to be present in the vermal B zone (Andersson and Oscarsson, 1978) and in the paravermal C1 and C3 zones (Ekerot and Larson, 1982), both of which receive somatosensory information. Neighboring IO neurons also have synchronized activity through electrotonic coupling, which results in synchronous complex spike activity in PCs arranged in a narrow longitudinal area (Sasaki et al., 1989; Lang et al., 1999). These PCs may also have synchronized simple spike activity (Wise et al., 2010). A similar organization is thought to be present throughout the cerebellar cortex and to represent the basic operational unit of the cerebellum (Apps and Hawkes, 2009). Since PCs in a narrow longitudinal band-shaped area project to the same target area in the DCN (above), some DCN neurons should receive input from multiple PCs that are synchronized. Synchronous input from PC axons causes time-locked activity in DCN neurons (Person and Raman, 2011). Thus, local branching of OC axons contributes to longitudinal functional organization of the cerebellar cortex.

### Transverse branching

Transverse branching seems to occur only in particular areas, mainly in the pars intermedia and vermis (above). Thus, the transverse branching does not seem to represent the general projection pattern of OC axons. Transverse branching of OC axons makes multiple longitudinal narrow areas that are separated mediolaterally, receive the same OC input, and project to the similar subarea in the DCN. Functional significance of this organization has not been much clarified. Further studies, including comparative ones, may be required to better characterize the transverse branching.

### Longitudinal branching

The most frequently observed category of longitudinal branching is the one across crus I in the pars intermedia and hemisphere. Branching between lobule V and paramedian lobule in C1 zone in cats (above) belongs to this category. Branching between lobules II–V and the copula pyramidis and branching between the simple lobule and the crus II-paramedian lobule, which are the most common pattern in the rat (Sugihara et al., 2001), also belong in this category. We think these branching patterns are not essentially different between cats and rats, since the copula pyramidis in the rat is equivalent with the caudal part of the paramedian lobule in the cat. This type of longitudinal branching fits well with the double localization of forelimb and hindlimb representation in the rostral and caudal pars intermedia in a classic study of evoked field potentials (Snider, 1950). A human imaging study also located arm and leg representation in lobules III–VI and in lobules VII–VIII in the pars intermedia and hemisphere (Timmann et al., 2009). CF input as well as MF input are involved in forming these representations. These rostral and caudal somatosensory areas are involved in different aspects of movement control (Timmann et al., 2009).

Furthermore, the longitudinal branching across crus I or lobule VIc represents a general organization of the entire cerebellar cortex, since longitudinal branching is seen in not only the somatosensory-responsive areas in the pars intermedia including C1 and C3 zones, but also in the rest of the cerebellum. MF axons also often make longitudinal branching across crus I and lobule VIc, projecting to both the rostral and caudal cerebellum simultaneously (Quy et al., 2011). Therefore, there seems to be general divergent organization in the afferent projection between the rostral and caudal cerebellum across crus I and lobule VIc. Thus, corresponding areas in the rostral and caudal cerebellum can belong to the same module. Yet, beyond generating a somatosensory double representation, the functional significance of the longitudinal branching remains unclear. Moreover, functional significance of the somatosensory double representation itself remains unclear, besides the fact that the rostral and caudal areas have different activities in somatosensory tasks (Timmann et al., 2009).

## Developmental basis for branching patterns of OC axons

Projections of CF and MFs begin to form in the late embryonic stages around embryonic days 14–18 (E14–E18) in the mouse (Sotelo et al., 1984; Ashwell and Zhang, 1992; Paradies and Eisenman, 1993). Although these projections have compartment-specific topography in adult (Sugihara and Shinoda, 2004, 2007; Quy et al., 2011), and the CF projection seems to have adult-like topography at P5 (Sugihara, 2005), it is not clear how precise a topography the immature climbing and MF projections have in the late embryonic stage. PCs are arranged in multiple clusters in the immature cerebellar cortex at this stage (Altman and Bayer, 1997). We have recently shown that the number of recognizable PC clusters is as large as 54 (Fujita et al., 2012). Therefore, we speculate that these PC clusters, which have different expression profiles of molecules, may form proper topographic connections in the cerebellar cortex through molecular matching between PCs and afferent axons during development (Sotelo, 2004). Neuronal clustering may be essential in the development of compartmentalization for topographic afferent and efferent connections as seen in the spinal cord (Sürmeli et al., 2011).

**Figure 5:**
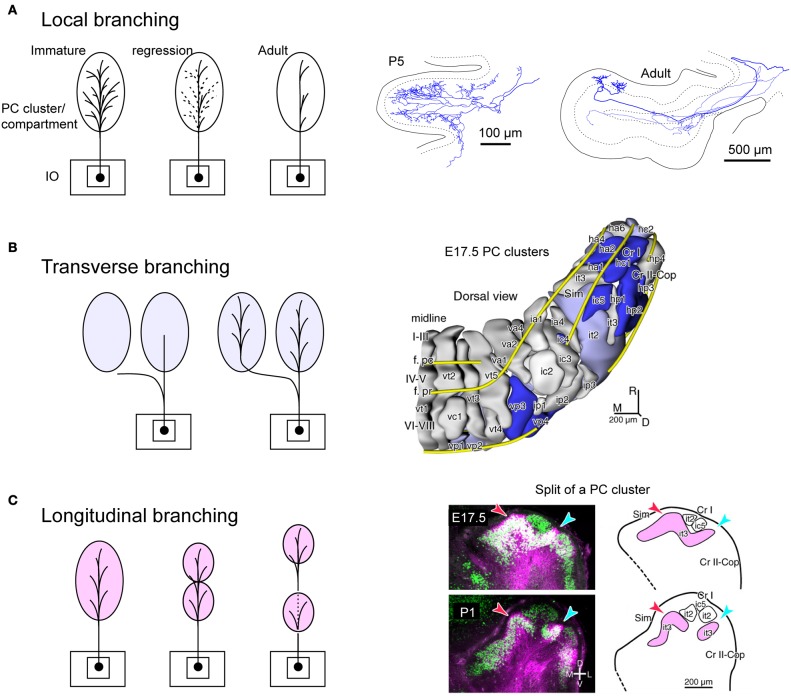
**Speculative illustration of formation of OC branching patterns. (A)** Projection by local branches to a narrow band-shaped subarea within a single aldolase C compartment may be formed in accordance with regression of exuberant branches. Right-hand insets show exuberant local branches in immature OC axons at P5 (cited from Sugihara, 2006). **(B)** Some transverse branching may be formed by branching of growing axon toward multiple clusters of PCs. Right-hand inset shows PC clusters in the E17.5 cerebellum (cited from Fujita et al., 2012). **(C)** Some longitudinal branching may be formed by longitudinal separation of a target compartment. Right-hand inset shows the longitudinal split of a PC cluster in the developing cerebellum (cited from Fujita et al., 2012). E17.5, embryonic day 17.5; P1 postnatal day 1; it2, ic5, it3, etc.; names of embryonic PC clusters.

We speculate that branching patterns of OC axons is hinted in the cluster organization of the immature cerebellar cortex. Each PC cluster has its own molecular expression profile (Wilson et al., 2011; Fujita et al., 2012), i.e., some molecules are expressed only in particular clusters of PCs. Eph receptors and cadherin family molecules are among these molecules (Nishida et al., 2002; Neudert et al., 2008; Hashimoto et al., 2012). Thus, these molecules are presumably involved in establishing correct OC connections. This may indicate that the embryonic OC projection, including all branches, may have precision to the level of clusters. A cluster is basically equivalent to a single molecular compartment in adulthood, since each embryonic PC cluster basically develops into single molecular compartment (Fujita et al., 2012). Immature OC axons have superabundant number of branches, which regress in the early postnatal period (Mariani and Changeux, 1981; Sugihara, 2005). It may be speculated that this regression process is involved in restricting the final local branching distribution to the microzone range, which is much narrower than a single molecular compartment (Figure 5A). Concerning transverse branching, supposing two mediolaterally separate clusters have similar molecular expression profiles, an OC axon may project to these two clusters by mediolateral branching (Figure 5B).

In relation to the longitudinal branching across crus I (see above), our analysis of PC cluster development has shown some clusters are “split” into rostral and caudal clusters underneath other clusters in crus I (Fujita et al., 2012). Supposing the initial cluster receives multiple branches of an OC axon, the splitting of this cluster would explain the final longitudinal branching pattern of OC axons and hence general mirror-image organization of the OC projection across crus I (Figure 5C). Thus, we hypothesize that crus I is the key lobule that determines the rostro-caudal arrangement of the cerebellum. Further studies on the development of PC clusters in the embryonic cerebellum will be required to clarify whether splitting of embryonic clusters can also explain other types of longitudinal and/or transverse branching.

## Conclusion

Branching patterns of OC axons can be classified into local, transverse, and longitudinal types. These branching patterns of OC axons are tightly related with the functional organization of the cerebellar cortex. The local branching is in accordance with the microzonal organization of the cerebellar cortex. The transverse and longitudinal branching patterns are in accordance with the compartmental organization of the cerebellar cortex and related to the lobular organization. The longitudinal branching, in particular, is a basis of the mirror-imaged double somatotopic representation of the body in the cerebellum. Attempts to relate the OC branching patterns to development of cerebellar compartmentalization and lobulation may be helpful in untangling the complicated compartmental and lobular organization of the cerebellar cortex.

### Conflict of interest statement

The authors declare that the research was conducted in the absence of any commercial or financial relationships that could be construed as a potential conflict of interest.
